# Utilization of Gallium-68 Fibroblast Activation Protein Inhibitor Positron Emission Tomography (⁶⁸Ga-FAPI PET) for Head and Neck Malignancies With Neck Imaging Reporting and Data System (Ni-RADS) Correlation

**DOI:** 10.7759/cureus.78476

**Published:** 2025-02-04

**Authors:** Revanth R Bhat, Shivakumar Swamy Shivalingappa, Mahesh Ashok, Avinash Kesari, Sumana Kedilaya, Yashas Ullas L

**Affiliations:** 1 Department of Radiology, Healthcare Global Hospital, Bengaluru, IND; 2 Department of Radiodiagnosis, Sri Devaraj Urs Medical College, Kolar, IND

**Keywords:** fapi, fapi pet-ct, head and neck cancers, nirads, pet ct scan

## Abstract

Background

Head and neck cancers (HNCs) encompass a group of malignancies that arise in the mucosal surfaces of the oral cavity, pharynx, larynx, and other related structures. Advances in imaging modalities such as positron emission tomography-computed tomography (PET-CT) and magnetic resonance imaging (MRI) have improved tumor detection and staging, aiding in personalized treatment approaches.

PET-CT is used to diagnose and stage various cancers. Interpretation of neck masses can be quite challenging, particularly in the context of prior surgery and radiotherapy.

A standardized lexicon and risk classification system for interpreting images in patients treated for HNC has been developed by the American College of Radiology (ACR) Neck Imaging Reporting and Data System (Ni-RADS) Committee. While fluorine-18 fluorodeoxyglucose (18F-FDG) PET-CT is widely used to assess head and neck malignancies, we have chosen to employ gallium-68 fibroblast activation protein inhibitor (^68^Ga FAPI) PET-CT in the Ni-RADS category in this investigation because to its exclusive advantages over FDG.

Methodology

This was a non-funded retrospective-prospective study conducted in the Department of Radiodiagnosis at Healthcare Global Hospital, KR Road, Bangalore, following approval from the Institutional Ethical Committee. The period of observation for this study was January 2024 to June 2024.

Patients with known HNC who were on follow-up and referred for a ^68^Ga FAPI PET-CT scan were included in the study. The Ni-RADS score was assigned, and histopathological correlation was performed. Descriptive statistics were used, and sensitivity and specificity were calculated.

Results

Out of the 41 cases selected for the study, all Ni-RADS 1 cases (100%, 6/6) were nonmalignant. Among Ni-RADS 2 cases 3 (37.5%) were malignant and 5 (62.5%) were nonmalignant. Nearly all Ni-RADS 3 cases (26/27, 96.3%) were malignant, indicating that higher Ni-RADS scores strongly correlate with malignancy. Recurrence is significantly associated with a Ni-RADS 3 score, whereas nonmalignancy is associated with a lower score (*P *< 0.001). Similar results were also seen in the case of nodal recurrences.

This study showed that ^68^Ga FAPI PET-CT has a high sensitivity of 88.3% and specificity of 95.8% in identifying recurrent malignant and nonmalignant cases.

Conclusions

We can conclude that FAPI PET, which offers several advantages over FDG, can be effectively used in the Ni-RADS criteria for diagnosing HNC recurrences. The utilization of FAPI PET in conjunction with contrast-enhanced CT facilitates the identification of tumor morphological and metabolic features. However, further research and larger cohorts are needed to improve prediction accuracy and guide personalized treatment decisions.

## Introduction

Head and neck cancer (HNC) ranks seventh globally in terms of overall cancer incidence, fifth among men, and 12th among women [1]. Tumors of the oral cavity, nasopharynx, oropharynx, hypopharynx, and larynx are included in head and neck squamous cell carcinoma (HNSCC) [2]. Imaging modalities available for HNC include ultrasound (USG), computed tomography (CT), magnetic resonance imaging (MRI), positron emission tomography (PET), and radionuclide imaging; however, PET in conjunction with CT (PET-CT) is used to diagnose and stage cancer, where CT helps evaluate the anatomy and PET is primarily used to assess physiology [3].

Interpreting neck masses can be exceedingly challenging, particularly if HNC treatment has been administered previously. The American College of Radiology Neck Imaging Reporting and Data System (Ni-RADS) has formulated recommendations for a standardized lexicon and risk assessment method to be applied in interpreting images in patients treated for HNC. Ni-RADS aids in the risk categorization of different imaging results and guides management decisions for the radiologist and clinical team [4]. Post-treatment lesions are characterized using different criteria for fluorodeoxyglucose (FDG) PET and CT results within the Ni-RADS category. However, in recent years, articles on gallium-68 fibroblast activation protein inhibitor (^68^Ga FAPI) have been published at an accelerating pace, and a new era in molecular imaging for both cancer and non-cancer conditions has been ushered in by FAPI imaging [5].

Several investigations have shown that ^68^Ga-FAPI can be beneficial for image-guided intervention, metastasis detection, and the diagnosis and differentiation of primary cancers. Its indications and therapeutic effects, however, are still being determined [6]. This choice was made to assess the diagnostic performance of ^68^Ga-FAPI PET-CT as opposed to FDG PET-CT in the Ni-RADS categorization of HNCs.

## Materials and methods

This was a non-funded, retrospective, and prospective study conducted in the Department of Radiodiagnosis at Healthcare Global Hospital, KR Road, Bangalore, following approval from the Institutional Ethics Committee. The study period was from January 2024 to June 2024. Siemens Biograph (Erlangen, Germany) and GE Discovery CT (Chicago, IL) machines were used for the study.

The study included patients with known cases of HNCs on follow-up, who were referred for FAPI PET-CT scans to the Department of Radiodiagnosis after meeting the inclusion criteria.

Methodology

Patients with known cases of HNC underwent ^68^Ga FAPI PET-CT scans and were assigned Ni-RADS scores. Histopathological correlation was subsequently performed, and the results were tabulated.

Ni-RADS scores are given as follows: The primary tumor site and neck lymph nodes are scored separately based on imaging suspicion of recurrence. The primary designator *X* can be used for an unknown primary. Ni-RADS 0: incomplete (prior imaging unavailable, but will be obtained), Ni-RADS 1: no evidence of recurrence, Ni-RADS 2: low suspicion of recurrence (2a: superficial mucosal, 2b: deep), Ni-RADS 3: high suspicion of recurrence, Ni-RADS 4: known recurrence.

Statistical analysis

The obtained results were statistically analyzed as described, and numerical and percentage summaries of qualitative data are included in the current investigation. The relationship between two qualitative variables was ascertained using the chi-square test, with a 95% significance threshold. SPSS version 22 (IBM Corp., Armonk, NY) was used to analyze the data.

Inclusion criteria

Patients who were clinically suspicious for HNC recurrence or had known cases of HNC on follow-up were included in the study. HNCs include cancers of the tongue, oral cavity (including the floor of the mouth and buccal mucosa), lip, pharynx, larynx, hypopharynx, palate, and other related structures.

Exclusion criteria

Patients on immunotherapy were excluded from the study.

Sample size calculation

Based on the prevalence of HNCs in India, with a confidence level of 90% and error margins of ±13%, we used the following formula in LaTeX built-in format to calculate the sample size of 41:

 $\text{Sample size} = \frac{\frac{z^2 \cdot p \cdot (1 - p)}{e^2}}{1 + \left(\frac{\frac{z^2 \cdot p \cdot (1 - p)}{e^2}}{N}\right)}$

where *N* is the population size; *e* the margin of error (percentage in decimal form, e.g., 0.05 for 5%), *z* = *Z *-score corresponding to the desired confidence level; and *p* the proportion of the population with the characteristic of interest (e.g., 0.5 if unknown).

## Results

The details of the gender-wise distribution of the cases are shown in Table 1.

**Table 1:** Details of gender-wise distribution of cases (N = 41). The data show a gender distribution where 78% (32/41) of participants were male and 22% (9/41) were female. This indicates a significant male majority in the sample, with males constituting over three-quarters of the total population.

	Frequency	Percentage
Female	9	22.0
Male	32	78.0
Total	41	100.0

The details of the sites of cancer are shown in Table 2.

**Table 2:** Frequency table of sites of cancer (N = 41). The data present the distribution of carcinoma cases across different sites among 41 patients. The most common site was the buccal mucosa, accounting for 15 cases (36.6%). Carcinoma of the tongue was the second most prevalent, with 12 cases (29.3%). The larynx was affected in 8 cases (19.5%). Less common sites included the oropharynx (2 cases, 4.9%), upper lip, floor of mouth, hard palate, and hypopharynx, each constituting 1 case (2.4%) of the total. This indicates that the buccal mucosa and tongue are the primary sites for carcinoma in this sample, while other sites are less frequently affected.

Tumor site	Frequency	Percentage
Buccal mucosa	15	36.6%
Carcinoma tongue	12	29.3%
Larynx	8	19.5%
Oropharynx	2	4.9%
Carcinoma upper lip	1	2.4%
Floor of mouth	1	2.4%
Hard palate	1	2.4%
Hypopharynx	1	2.4%
Total	41	100.0

The details of the Ni-RADS score of the subjects are given in Table 3.

**Table 3:** Distribution of overall Ni-RADS scores in patients (N = 41). The table presents the distribution of overall Ni-RADS scores among 41 patients. Ni-RADS 1, indicating low suspicion for recurrence, was observed in 6 patients (14.6%). Ni-RADS 2, representing moderate suspicion, was seen in 8 patients (19.5%). The majority of patients (27, 65.9%) were classified as Ni-RADS 3, which signifies high suspicion of recurrence. This distribution highlights that a substantial proportion of the patient cohort falls into the high-risk category for recurrence. Consequently, these patients require a biopsy.

Overall Ni-RADS	Frequency	Percentage
1	6	14.6%
2	8	19.5%
3	27	65.9%
Total	41	100.0%

The details of the histopathology of the subjects are given in Table 4.

**Table 4:** Distribution of histopathology results in patients (N = 41). The table outlines the histopathology results for 41 patients. The majority of patients (27, 65.9%) were diagnosed with squamous cell carcinoma (SCC). Cases with no malignancy accounted for 11 patients (26.8%) of the total cohort. Other diagnoses included adenoid cystic carcinoma (1, 2.4%), metastatic SCC (1, 2.4%), and osteoradionecrosis (1, 2.4%). This distribution indicates that SCC is the predominant histopathological finding, emphasizing its prevalence in this cohort.

Histopathology	Frequency	Percentage
Adenoid cystic carcinoma	1	2.4%
Metastatic SCC	1	2.4%
No malignancy	11	26.8%
Osteoradionecrosis	1	2.4%
SCC	27	65.9%
Total	41	100.0%

The details of the association between histopathology outcomes and Ni-RADS scores are given in Table 5.

**Table 5:** Details of the association between histopathology outcomes and Ni-RADS scores (N = 41). *Chi-square test applied (*P* = 0.0000012). All Ni-RADS 1 cases (6/6, 100%) were nonmalignant. Ni-RADS 2 cases were 37.5% malignant and 62.5% nonmalignant. Nearly, all Ni-RADS 3 cases (26/27, 96.3%) were malignant, indicating higher Ni-RADS scores strongly correlate with malignancy. Recurrence is significantly associated with Ni-RADS 3 score, whereas nonmalignancy is associated with lower scores (*P *< 0.001). Ni-RADS, Neck Imaging Reporting and Data System

Histopathology with overall Ni-RADS	Ni-RADS 1		Ni-RADS 2		Ni-RADS 3		Total
	n	%	n	%	n	%	
Malignant	0	0.0%	3	37.5%	26	96.3%	29
Nonmalignant	6	100.0%	5	62.5%	1	3.7%	12
Total	6	100.0%	8	100.0%	27	100.0%	41

**Figure 1:**
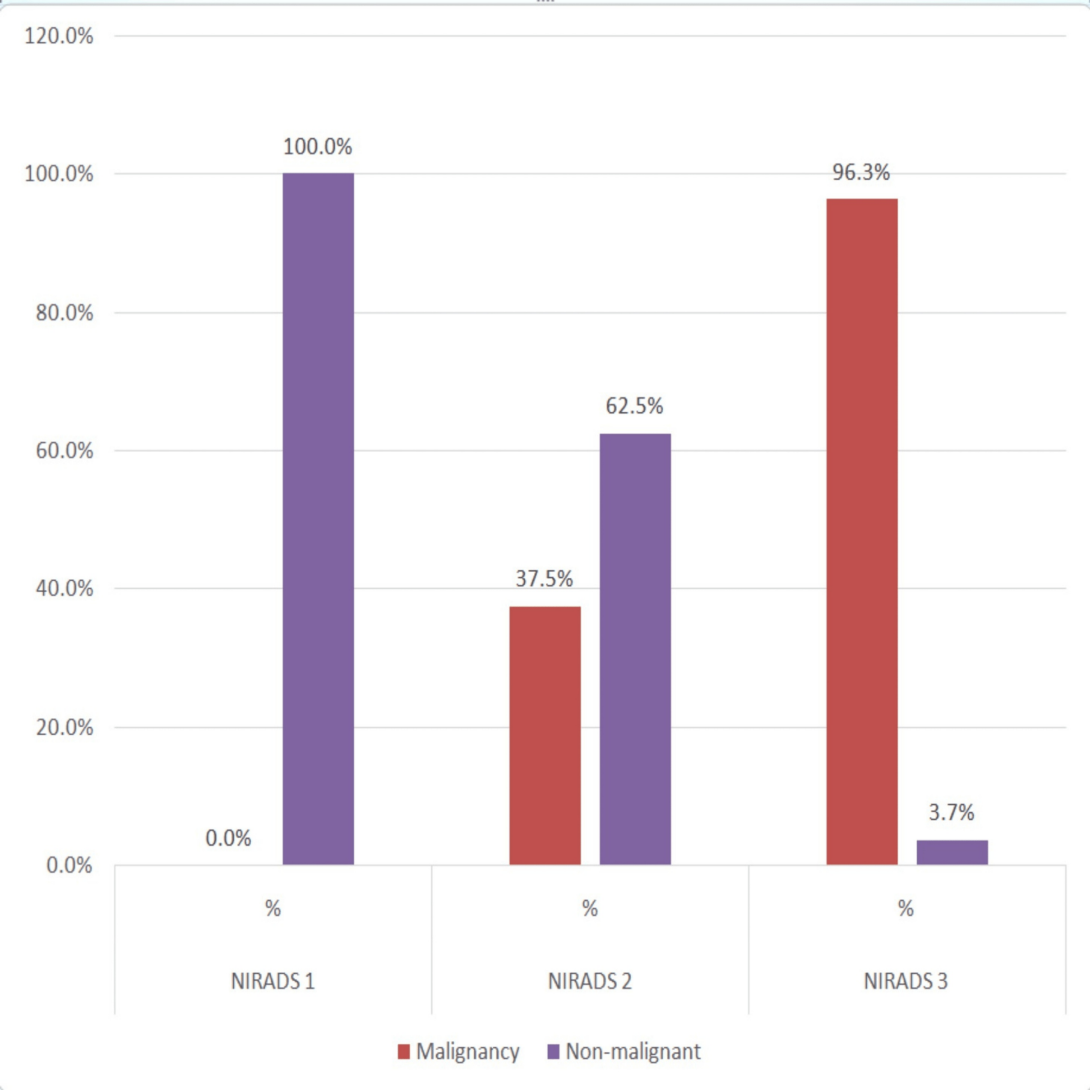
Details of the association between histopathology outcomes and Ni-RADS scores (N = 41). Ni-RADS, Neck Imaging Reporting and Data System

Graphical representation of the association of histopathology outcome with Ni-RADS score is shown in bar chart format in Figure 1.

Details of the sensitivity and specificity values for overall Ni-RADS ratings, considering histopathology as the gold standard, are shown in Table 6.

**Table 6:** Details of the sensitivity and specificity values for overall Ni-RADS ratings, considering histopathology as the gold standard. *Chi-square test applied (*P*-value = 0.0000012). This study shows that FAPI PET has a high sensitivity of 89.6% and a specificity of 91.6% in identifying recurrent malignant and nonmalignant cases. This implies that FAPI PET is effective in detecting malignancies while accurately excluding nonmalignant cases. FAPI, fibroblast activation protein inhibitor; PET, positron emission tomography; Ni-RADS, Neck Imaging Reporting and Data System

	Sensitivity	Specificity
FAPI PET	89.6%	91.6%

According to the Ni-RADS classification, lymph nodes are categorized into different Ni-RADS categories. Table 7 presents the distribution of lymph nodes across these categories.

**Table 7:** Distribution of overall Ni-RADS scores for lymph nodes (N = 35). The overall Ni-RADS score was not applicable in six patients. Among the 35 cases, 51% were rated as Ni-RADS 1, 11% as Ni-RADS 2, and 37% as Ni-RADS 3. Ni-RADS, Neck Imaging Reporting and Data System

Overall Ni-RADS	Frequency	Percentage
1	18	51%
2	4	11%
3	13	37%
Total	35	100%

Table 8 shows the association between node histopathology and overall Ni-RADS ratings.

**Table 8:** Association between nodal histopathology and overall Ni-RADS ratings (N = 35). Chi-square test applied (*P*-value < 0.001). The table compares node histopathology with overall Ni-RADS ratings in terms of percentages. For Ni-RADS 1, 1 lymph node (5.6%) was positive and 16 (94.4%) were negative. For Ni-RADS 2, 1 (25%) was positive and 3 (75%) were negative. For Ni-RADS 3, all 13 (100%) were positive and 0% was negative. This shows a clear trend: as the Ni-RADS score increases, the percentage of positive histopathology also increases, from 5.9% in Ni-RADS 1 to 100% in Ni-RADS 3. Conversely, the percentage of negative histopathology decreases from 94.1% in Ni-RADS 1 to 0% in Ni-RADS 3. This highlights a strong correlation between higher Ni-RADS ratings and positive node histopathology, which is statistically significant (*P*-value < 0.001). Ni-RADS, Neck Imaging Reporting and Data System

Node histopathology	Overall Ni-RADS						Total
	1		2		3		
	n	%	n	%	n	%	
Positive	1	5.6%	1	25%	13	100%	15
Negative	17	94.4%	3	75%	0	0%	20
Total	18	100%	4	100%	13	100%	35

Table 9 presents the distribution of sensitivity and specificity values for overall Ni-RADS ratings, considering node histopathology as the gold standard.

**Table 9:** Details of the sensitivity and specificity values for overall Ni-RADS ratings, considering node histopathology as the gold standard. Chi-square test applied (*P*-value < 0.001). This study shows that FAPI PET has a high sensitivity of 87% and 100% specificity in identifying malignant and nonmalignant lymph nodes. FAPI, fibroblast activation protein inhibitor; PET, positron emission tomography; Ni-RADS, Neck Imaging Reporting and Data System

Node histopathology	Sensitivity	Specificity
FAPI PET	87%	100%

Table 10 shows us the overall sensitivity and specificity of the study. 

**Table 10:** FAPI PET's overall sensitivity and specificity in identifying head and neck cancers. Chi-square test applied (*P*-value < 0.001). According to this study, FAPI PET can distinguish between malignant and nonmalignant patients with a high sensitivity of 88.3% and a specificity of 95.8%. This suggests that FAPI PET is effective in identifying malignancy and ruling out noncancerous conditions. FAPI, fibroblast activation protein inhibitor; PET, positron emission tomography

Node histopathology	Sensitivity	Specificity
FAPI PET	88.3%	95.8 %

Case 1

**Figure 2:**
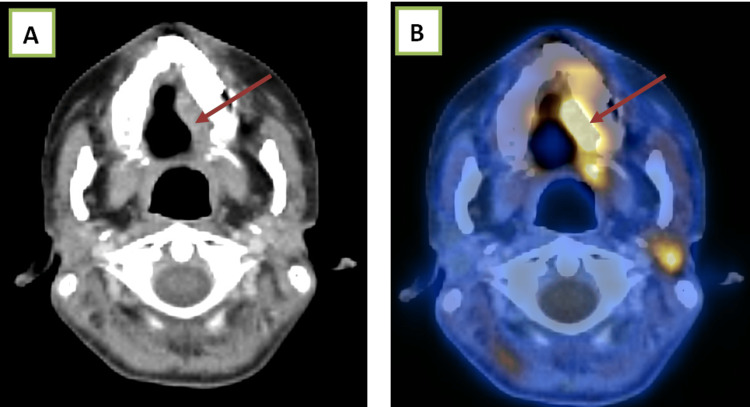
(A) Contrast-enhanced computed tomography and (B) fused PET-CT images show soft tissue thickening and heterogeneous enhancement along the left lateral border of the hard palate, with significant uptake on the fused PET-CT image. A heterogeneously enhancing left level II lymph node with significant Ga-FAPI uptake is also observed. Ga-FAPI, gallium fibroblast activation protein inhibitor; PET-CT, positron emission tomography-computed tomography

A 60-year-old male with carcinoma of the hard palate on follow-up, showing a Ni-RADS 3 lesion (Figure 2).

Case 2

**Figure 3:**
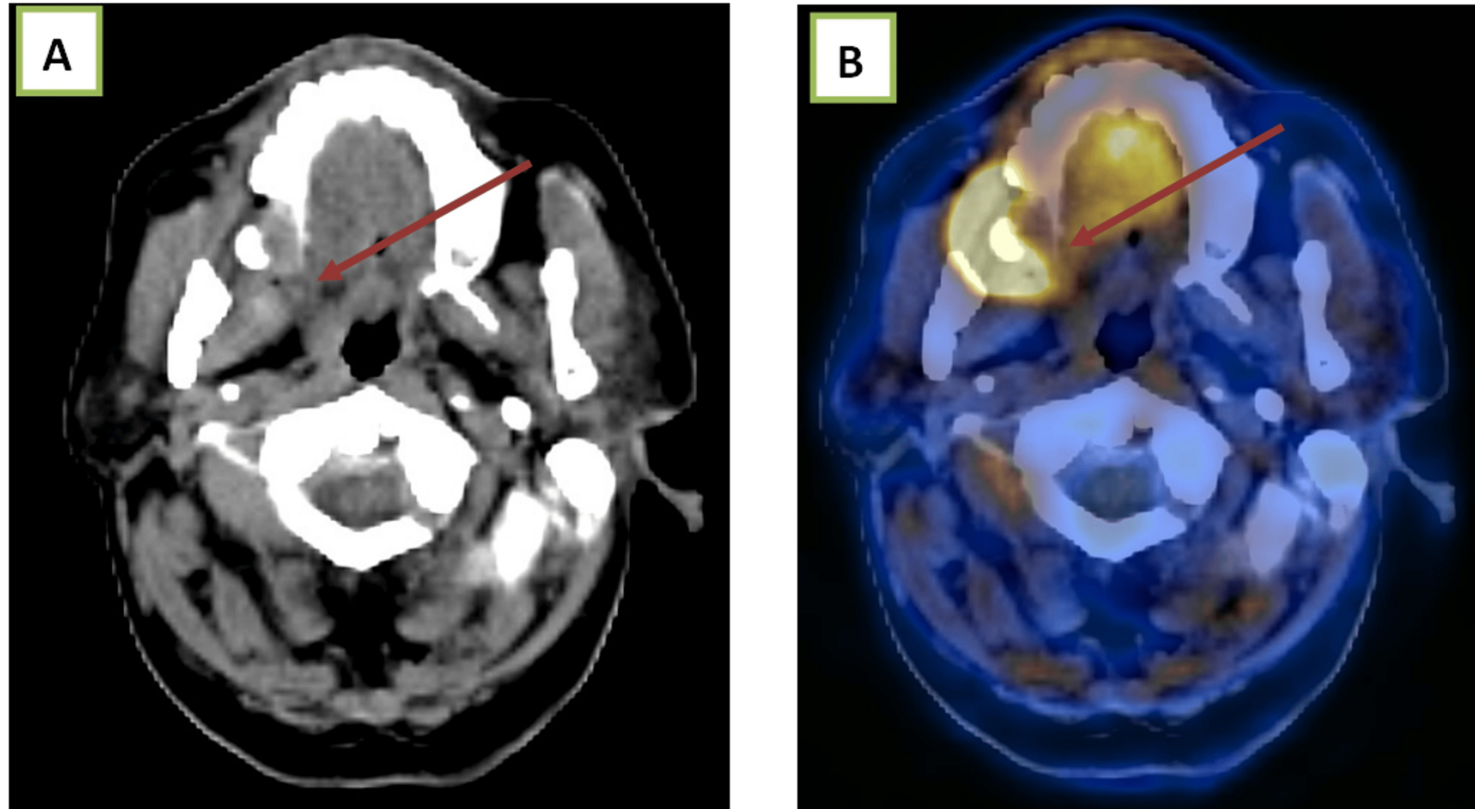
(A) Contrast-enhanced CT and (B) fused PET-CT images show a heterogeneously enhancing lesion in the right retromolar trigone and hard palate, with significant uptake on the fused PET-CT image. PET-CT, positron emission tomography-computed tomography

A 54-year-old female with carcinoma of the retromolar trigone on follow-up, showing a Ni-RADS 3 lesion (Figure 3).

Case 3

**Figure 4:**
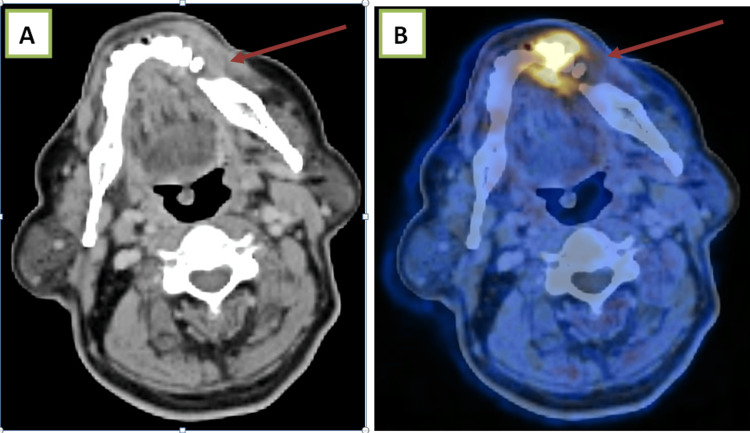
(A) Contrast-enhanced CT and (B) fused PET-CT images show recurrent soft tissue thickening and adjacent osseous erosion. PET-CT, positron emission tomography-computed tomography

Recurrent carcinoma of the left buccal mucosa with osseous erosion, showing a Ni-RADS 3 lesion (Figure 4).

Case 4

**Figure 5:**
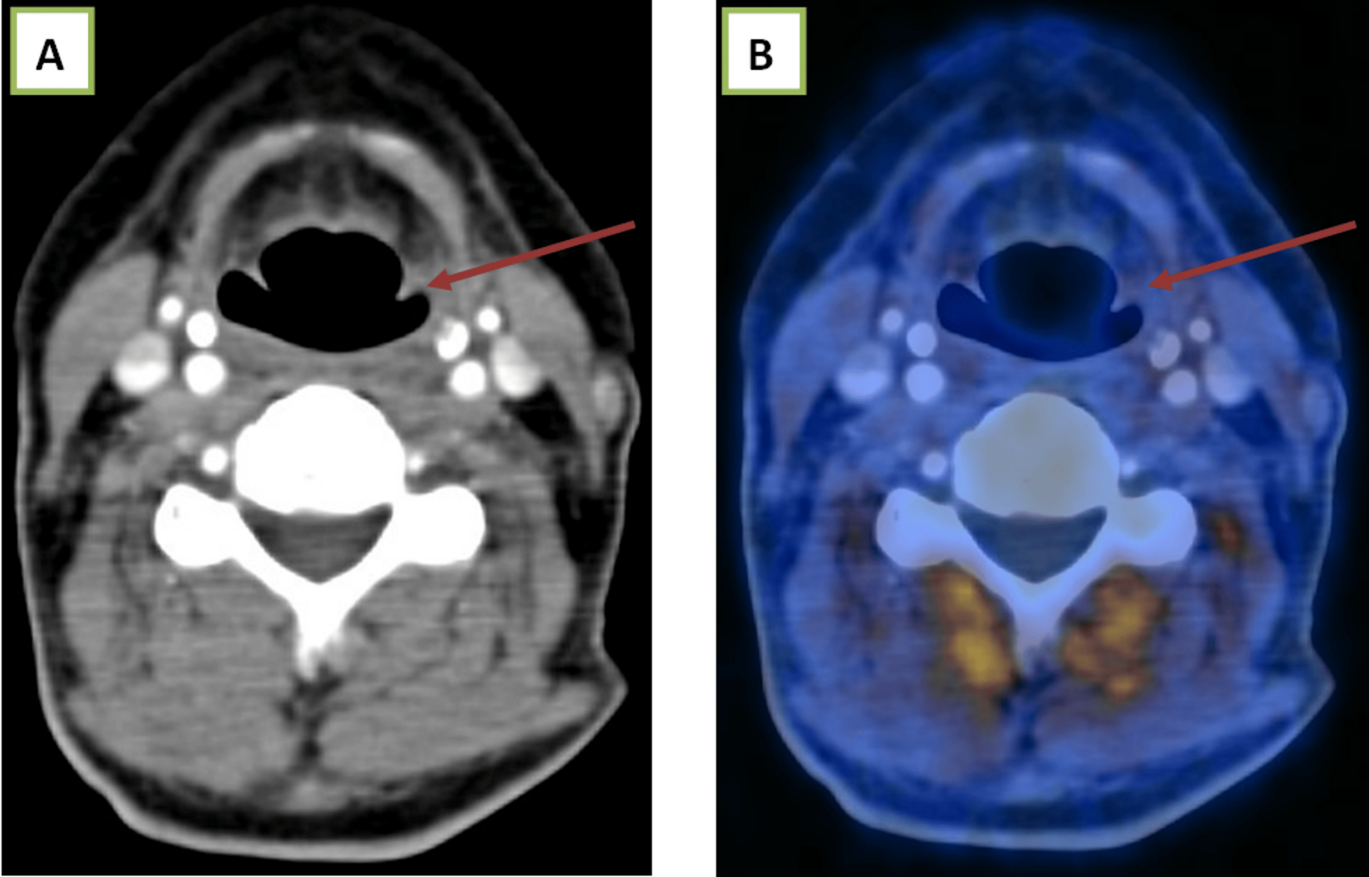
(A) Contrast-enhanced CT and (B) fused PET-CT images show mild thickening of the left aryepiglottic fold with no significant uptake on the fused images, corresponding to a Ni-RADS 1 category. PET-CT, positron emission tomography-computed tomography; Ni-RADS, Neck Imaging Reporting and Data System

Case of carcinoma of the left aryepiglottic fold on follow-up, showing a Ni-RADS 1 lesion (Figure 5).

## Discussion

In our study, out of 41 cases, 32 were males (78%) and 9 were females (22%), which was in accordance with the existing gender prevalence of HNCs. According to a retrospective study by Park et al., males are significantly more likely than females to develop HNC, regardless of tobacco or alcohol consumption [7].

The most common site in this study was the buccal mucosa, accounting for 15 cases (36.6%). Carcinoma of the tongue was the second most prevalent, with 12 cases (29.3%). The larynx was affected in 8 cases (19.5%). Less common sites included the oropharynx (2 cases, 4.9%), upper lip, floor of the mouth, hard palate, and hypopharynx, each constituting 2.4% (1 case) of the total. This is in line with the National Cancer Registry, which reports that buccal mucosa cancer is the most prevalent type of HNC among the people of India [8].

In our study, the majority of patients, 65.9% (27 patients), were diagnosed with SCC, with adenoid cystic carcinoma and metastatic SCC being the other histological variants. This is consistent with the fact that squamous cell carcinoma accounts for more than three-fourths of HNCs [9].

Among the 41 patients included in our study, recurrent malignancy was identified in 29 patients (70.7%), indicating that the majority of the cases were cancerous. Nonmalignant findings were observed in 2 patients (29.3%). All Ni-RADS 1 cases (6/6, 100%) were nonmalignant. Ni-RADS 2 cases were 37.5% malignant and 62.5% nonmalignant. Nearly all Ni-RADS 3 cases (26/27, 96.3%) were malignant, indicating higher Ni-RADS scores strongly correlate with malignancy. Recurrence was significantly associated with a Ni-RADS 3 score, whereas nonmalignancy was associated with a lower score. However, increased FAPI activity was observed in a case of osteoradionecrosis, leading to a false positive result.

Among the 41 patients included in our study, Ni-RADS 1 was the most common, observed in 45% (18 patients). Ni-RADS 3 was seen in 32.5% (13 patients), Ni-RADS 2 in 10% (4 patients), and 14.6% (6 patients) had no node involvement. On histopathological analysis, among Ni-RADS 1 lymph nodes, 1 lymph node (5.6%) was positive and 16 (94.4%) were negative. For Ni-RADS 2, 1 (25%) was positive and 3 (75%) were negative. For Ni-RADS 3, all 13 (100%) were positive and 0% was negative. This shows a clear trend: as the Ni-RADS score increases, the percentage of positive histopathology also increases, from 5.9% in Ni-RADS 1 to 100% in Ni-RADS 3. This highlights a strong correlation between higher Ni-RADS ratings and positive node histopathology, which is significant statistically (*P*-value < 0.001).

As per this study, FAPI PET had a high sensitivity of 88.3% and 95.8% specificity in identifying malignant and nonmalignant cases. This indicates that FAPI PET is effective in detecting malignancies while accurately excluding nonmalignant cases.

Various other studies have also shown that Ni-RADS categorization has high sensitivity and specificity in diagnosing recurrent HNCs. Kumar et al. reported that disease persistence rates for the main tumor location were 4% for Ni-RADS 1, 24% for Ni-RADS 2, and 80% for Ni-RADS 3 scores. Ni-RADS categories 1, 2, and 3 for lymph nodal evaluation showed nodal disease recurrence rates of 5.3%, 25%, and 66.7% [10].

In another study by Krieger et al., Ni-RADS performed well, showing a noteworthy difference in illness rates between groups: 3.8% for Ni-RADS 1, 17.2% for Ni-RADS 2, and 59.4% for Ni-RADS [11]. Hence, Ni-RADS criteria can be used as a good predictor of recurrent HNCs.

Targeting fibroblast activation protein (FAP) has been made possible by FAPI PET/CT, a novel medical imaging approach. There are several advantages to this new method, including decreased background noise and enhanced tumor absorption. Consequently, excellent tumor signal-to-background ratios are seen in FAPI PET/CT images, enabling accurate tumor staging, characterization, and detection [12].

FAPI PET-CT has several advantages over F18-FDG PET-CT. The tracer activity is not dependent on blood sugar levels, so overnight fasting is not required. Additionally, physical activity and muscle uptake related to high blood glucose are not present on FAPI. No brain or heart background activity is observed, and the synthesis of FAPI requires gallium generators rather than a cyclotron.

According to two studies by Kömek et al. and Gündoğan et al., ^68^Ga-FAPI is superior to 18F-FDG in determining the tumor stage and identifying lymph nodes, as well as detecting distant metastases [13,14].

In detecting primary staging and non-primary tumor metastasis of abdominopelvic malignancies, ^68^Ga-FAPI and 18F-FDG PET/CT demonstrated high overall diagnostic performance, according to a meta-analysis by Liu et al. However, the detection ability of ^68^Ga-FAPI was significantly higher than that of 18F-FDG [5].

In patients of head and neck malignancies with unknown primary and negative 18F-FDG results, ^68^Ga-FAPI PET-CT may increase the primary tumor detection rate, according to research by Gu et al. Additionally, ^68^Ga-FAPI performed similarly to 18F-FDG in evaluating metastases [15].

According to another study by Dong et al., when compared to traditional imaging techniques, FAPI imaging may lead to fresh possibilities in oncological diagnosis and care. The inability of FAPI imaging to distinguish between inflammatory and malignant tissue has been observed to result in false-positive findings [16].

## Conclusions

According to our study, we can conclude that FAPI PET, which offers several advantages as listed over F18-FDG, can be used in Ni-RADS criteria for diagnosing HNC recurrences effectively.

The investigation yielded data with a sensitivity of 88.3% and specificity of 95.6% for the detection of recurrent HNCs. The combination of FAPI PET with contrast-enhanced CT helps us to recognize the morphological and metabolic characteristics of tumors. Integrating these findings with histopathological data holds immense potential in refining diagnosis, treatment planning, and prognostic assessment. Further research, larger cohorts standardization of FAPI PET interpretation, and cost-effective analysis are needed to improve prediction accuracy and guide personalized treatment decisions.
